# Artificial Neural Network Modelling for Optimizing the Optical Parameters of Plasmonic Paired Nanostructures

**DOI:** 10.3390/nano12010170

**Published:** 2022-01-04

**Authors:** Sneha Verma, Sunny Chugh, Souvik Ghosh, B. M. Azizur Rahman

**Affiliations:** 1 School of Mathematics, Computer Science and Engineering, City University of London, London EC1V 0HB, UK; Sunny.Chugh.3@city.ac.uk (S.C.); b.m.a.rahman@city.ac.uk (B.M.A.R.); 2 Department of Electronics and Electrical Engineering, University College London, London EC1V 0HB, UK; souvik.ghosh@ucl.ac.uk

**Keywords:** nano structures, sensitivity, Q-factor, plasmonic wavelength, full-width half maximum (FWHM), artificial neural networks (ANNs), machine learning (ML), hidden layers and neurons

## Abstract

The Artificial Neural Network (ANN) has become an attractive approach in Machine Learning (ML) to analyze a complex data-driven problem. Due to its time efficient findings, it has became popular in many scientific fields such as physics, optics, and material science. This paper presents a new approach to design and optimize the electromagnetic plasmonic nanostructures using a computationally efficient method based on the ANN. In this work, the nanostructures have been simulated by using a Finite Element Method (FEM), then Artificial Intelligence (AI) is used for making predictions of associated sensitivity *(S)*, Full Width Half Maximum (FWHM), Figure of Merit *(FOM)*, and Plasmonic Wavelength *(PW)* for different paired nanostructures. At first, the computational model is developed by using a Finite Element Method (FEM) to prepare the dataset. The input parameters were considered as the Major axis, *a*, the Minor axis, *b*, and the separation gap, *g*, which have been used to calculate the corresponding sensitivity (nm/RIU), FWHM (nm), FOM, and plasmonic wavelength (nm) to prepare the dataset. Secondly, the neural network has been designed where the number of hidden layers and neurons were optimized as part of a comprehensive analysis to improve the efficiency of ML model. After successfully optimizing the neural network, this model is used to make predictions for specific inputs and its corresponding outputs. This article also compares the error between the predicted and simulated results. This approach outperforms the direct numerical simulation methods for predicting output for various input device parameters.

## 1. Introduction

Nanostructures have recently gained a lot of attention from researchers because of their diverse applications, and the global market value of nanotechnology is predicted to reach USD 90 billion by 2021 [1] as consumer and industrial applications of nanostructures are rising continuously [2]. Simultaneously, Artificial Intelligence (AI) has also seen rapid growth in the last decade [3] and being adopted by computer scientists and specialists and by other researchers in various fields. It has shown widespread popularity in handling complex data-driven problems in science and technology [4]. These models have an unrivaled ability to identify and forecast patterns in data and identify unexpected trends that a human observer may be unable to recognize [5]. It can excel at detecting latent data structures and classifying extremely nonlinear datasets, making it suitable for many scientific methods. With the help of Machine Learning (ML), all researchers working on light-matter interaction have turned onto a new level, aided by materials science, physics, and photonic technologies. Two recent developments demonstrate this: The first is the emergence of intelligent photonic systems, another one is the integration of ML into physical and chemical sciences for in-depth knowledge acquisition and innovative fundamental insights [6].

Recently, photonics technology upgraded its functionality by enabling ML techniques and outperforms traditional photonics, which is poor in time/cost-ineffective and provided limited performance. Hence, many researchers have moved their focus to ML and used it in various applications such as coherent optical communication systems [7], plasmonics [8,9], multimode fibers [10,11], sensing [12,13,14,15,16], photonic crystal fibers [17], and nanotechnology [18,19,20,21,22]. Nanophotonics provides an excellent example of optical resonances and strong localized fields, which can be optimized by changing the geometry and material selection of the nanoparticles for various applications.

In this article, paired nanostructures have been considered and used to calculate the corresponding sensitivity, FWHM, FOM, and plasmonic wavelength. These paired nanostructures have been designed and simulated by using a finite element method (FEM). It is well known that rigorous full vectorial simulations of 3D photonic systems are time-consuming and demanding hours or even days to solve a complex system [23]. To overcome this issue, we have introduced a deep learning model designed on Python (version 3.8.3) framework, which proved to be quick in estimating. Deep artificial neural networks hold great potential to tackle complex problems in nano-optics, such as tailoring optical characteristics of single or paired nanostructures [24]. On the second phase, the neural network has been developed and trained with the help of the collected dataset obtained from the FEM. The labeled input parameters such as the Major axis, *a*, the Minor axis, *b*, the separation gap, *g*, and the height, *h*, are used to predict the following output parameters: sensitivity, FWHM, FOM, and plasmonic wavelength. To commence with the training of developed neural network, the learning algorithm generates an inferred function that predicts output values. This developed model can provide output parameters using any new input values after sufficient training. This learning algorithm can compare its predicted output with the actual output values and calculate the mean squared errors to show the accuracy of the designed model. In this deep neural network, many popular ML frameworks have been used while developing and training the network, such as *pandas* [25] for data cleaning, *Scikit-learn* [26], which is a higher-end library that is used for regression analysis, *NumPy* [27] used for multi-dimensional arrays and matrices, and *pickel* [28] used to serialize and deserialize a Python object structure. Finally, the *Pytorch* has been used [29,30,31], which is an ML package based on Torch tensors. It is a very popular open-source, developed by Facebook’s AI Research lab (FAIR) [32] in 2016 based on the programming language *Lua* [33], which is analogous to *NumPy* arrays, with an added benefit of GPU support. This is an important approach as it aids in accelerating numerical computations that can boost the speed of the neural network up to 50 times. It offers an easy-to-use API; thus, it is simple to interface with Python. The reason for the use of this brilliant platform is that it allows creating dynamic computing graphs that can be changed in real-time, which is required during the neural network training. *PyTorch* uses several back ends rather than a single back end for CPU, GPU, and other functional characteristics, so we have used FEM simulations in the back end for data collection, which is used for neural network training, and in the front end, *Pytorch* and *Scikit-learn* have been used due to their excellent imperative architecture which provides fast and lean approaches. This research creates the opportunity to calculate the optical parameters for paired nanostructural devices by using artificial neural network optimization methods.

## 2. The Convergence of Machine Learning with Nanostructural Devices

Finite element method based simulations were performed by using commercial software (COMSOL multiphysics 5.5), where paired gold nanoparticles were placed on a quartz substrate. In practice, a few tens of micrometres thick quartz substrate was used as a base of the antenna. Therefore, a 600 nm quartz substrate section was considered here. The use of PML boundaries truncates the computational domain. The dielectric constant of metal layer has been calculated with the help of the Drude free electron model because of the presence of the free-electron in the metal. In this model, the dielectric constant is calculated with the aid of relaxation time τ = 9.3 ± 0.9 $\times \;10^{-15}$ and for metals at near-infrared frequencies when ω>>1/τ,^34^]:

(1) $$\begin{array}{c} \varepsilon \left(\omega \right)=1-\frac{\omega_{p}{}^{2}}{\omega^{2}}+j\frac{\omega_{p}{}^{2}}{\omega^{3}\tau }=\varepsilon {}_{real}^{f}+j\varepsilon {}_{imag}^{f} \end{array}$$

**Figure 1:**
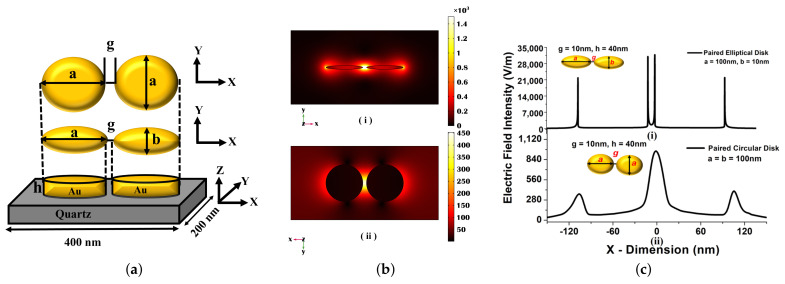
(**a**) Schematic of circular and elliptical nanostructure placed on quartz substrate. (**b**) Mode profile $E_{x}$ of paired gold (i) elliptical and (ii) circular nanoantenna. (**c**) The line plot of the electric field confinement at the separation gap and the corners of the nanoantenna.

Here, $\omega_{p}$ is the plasma angular frequency equal to $\sqrt{\frac{4\pi Ne^{2}}{m_{0}}}$ = 9 eV , and *N* and $m_{0}=0.99\pm 0.04$ are the conduction electron density and effective optical mass, respectively [34]. Figure 1a shows a conceptual and schematic representation of the paired nanostructures and its arrangement. We considered the nanoantenna on a quartz substrate. This configuration makes the whole structure more stable and easier to operate compared to scattered nanoparticles in a liquid medium. A quartz substrate is transparent to a broadband region and also chemically resistive. The height of the quartz substrate was considered as 600 nm and the height of the nanoantennas are independent of the substrate. A paired metal antenna was excited by x-polarized light in the z-direction from the top with wave excitation ON, and additionally, scattering boundary conditions (SBC) have been placed at the bottom and top of the computational domain. The entire numerical problem was solved in the frequency domain to obtain the scattered field distributions and the optical transmission/reflection spectra. In the FEM solver, the entire structure was discretised into ‘study’ mesh elemental size. Additionally, a 200 nm perfectly matched layer (PML) on the top of the air domain and bottom of the quartz substrate was introduced to avoid the artefacts of back reflection during computation. The unit cell dimension of the quartz substrate was kept fixed at 400 × 200 nm^2^, which is enclosed by a perfect electric conductor (PEC) and a perfect magnetic conductor (PMC) along x and y boundaries, respectively, to enforce the periodicity of the nanostructure. The height of the metal disks was taken as 40 nm throughout the work [35].

Figure 1b shows the variation of electric field $E_{x}$ in the x-direction through the centre of the pair of antennae, which is computed with the help of COMSOL Multiphysics, and it shows a very high electric field confinement in the separation region and the corners of the elliptical and circular antennae. Since electron conduction produces an efficient force at the surface of the paired structure this results in a substantial enhancement of electric field in the separation gap region, as seen in Figure 1c. Figure 1c(i) shows the maximum electric field for *a* = 100 nm, *b* = 10 nm, *g* = 10 nm, and *h* = 40 nm, the maximum electric field reaches up to 35,000 V/m at inner edge of the paired elliptical disks, and it can be observed that the maximum field intensity is 97.14% higher than the case of circular disk (shown in Figure 1c(ii)). From the electric field profile, it can be concluded that for a paired structure, a significant field enhancement can be observed at sharp corners by reducing the minor axis, *b*. The LSPR enhancement takes place as a result of this coupling, as the elliptical structures interact more firmly when they become closer to each other. The shift in optical transmission/reflection and absorption spectra of the resonating wavelength was smaller when the separation distance was higher, so in order to achieve a strong electric field confinement, a smaller separation distance is preferred [35].

After successfully developing the computational model, this approach was used to vary the major axis *a*, minor axis *b*, and separation distance *g* of the nanostructure and calculated their corresponding sensitivity *S*, which is defined as the ratio of resonant wavelength $\left(\lambda_{res}\right)$ shift with the change in the environmental refractive index $\delta n_{s}\left(\mathrm{RIU}\right)$, where $S=\delta \lambda_{res}\left(\mathrm{nm}\right)/\delta n_{s}\left(\mathrm{RIU}\right)$. As for a efficient sensor design, its sensitivity is the key objective, but the sharpness of the transmission and reflection curve is also important for their easy detection. As measurement accuracy also depends on the sharpness of the resonance curves and this can be quantified by the FWHM which is defined as the difference between the two wavelengths, i.e., $FWHM=\lambda_{1}-\lambda_{2}$, where $\lambda_{1}$ and $\lambda_{2}$ are the wavelengths, when the response is half of its maximum values. Additionally, FOM was considered as the third important output parameter, which was defined as the ratio of the sensitivity to the FWHM, i.e., $FOM=S(\mathrm{nm}\;{\mathrm{RIU}}^{-1})/FWHM$, and the plasmonic wavelength was considered as the fourth output parameter as it tells about the maximum relative response amplitude at particular wavelengths [35,36] to collect the dataset for neural network training. These rigorous simulations of the nanostructures were time-consuming and complex. To overcome this drawback of the direct numerical simulations, the time-efficient ML algorithm has been developed. We have focused on the number of input parameters that can help to train the neural network. Several simulations have been performed to collect the dataset for neural network training. The major axis *a*, minor axis *b*, and separation distance *g* were adopted as the input parameters, and the sensitivity, FWHM, FOM, and plasmonic wavelength were considered as the output parameters. Furthermore, with the help of a supervised learning algorithm, a neural network was developed to make the accurate predictions after learning through a training dataset. Different layers in feed-forward and back-propagation neural networks are structured in multi-layered perceptron (MLP) architectures that use the back-propagation (BP) algorithm and various activation functions. In this study, we have designed an algorithm that can be used for multi-input and -output systems and only need a one-time training process that requires few minutes to generate accurate output parameters. In this way, this algorithm can be beneficial for a similar nanoantenna design. Finally, this paper represents the forward modelling facilitated by neural networks, which has demonstrated the ability of the AI algorithm to learn complex relationships between nanophotonic structures and their related optical responses.

### 2.1. Artificial Neural Networks for Prediction of Output Parameters of Nanophotonic Structures

We live in an exciting era of technology, wherein ML has a significant impact on various applications, from big data handling to making accurate predictions. However, data gathering/collection can became a critical pitfall in the field of ML. The majority of the time spent running the ML algorithm from the starting point until the end is taken by the data preparation, including data collection, cleaning, evaluating, visualizing, and feature engineering. Hence, it becomes an important task to develop this ML algorithm. Furthermore, the outputs are based on the optimizations performed by the designed algorithm. After collecting the dataset, the developed algorithm performs a set of data analysis techniques. The task of the developed model on training data is automated with little or no human interventions. Hence, the importance of COMSOL multiphysics data analysis cannot be underestimated. ML is a significant contributor to facilitate the quick processing of a large amount of data generated by nanostructures to develop the valuable patterns for data scientists. Table 1 shows the variation of a data frame or a sequence of numeric values of the dataset, which were obtained from COMSOL multiphysics. As a consequence, the task of classifying data into different categories is entirely automated. The percentile, mean, standard deviation, minimum, and maximum have been calculated for the simulated data frame as shown in Table 1.

In Table 1, the dataset variation, and the relationships of the dataset along with its trends and patterns can be seen.

### 2.2. The Architecture of the Multilayer Artificial Neural Network

**Figure 2:**
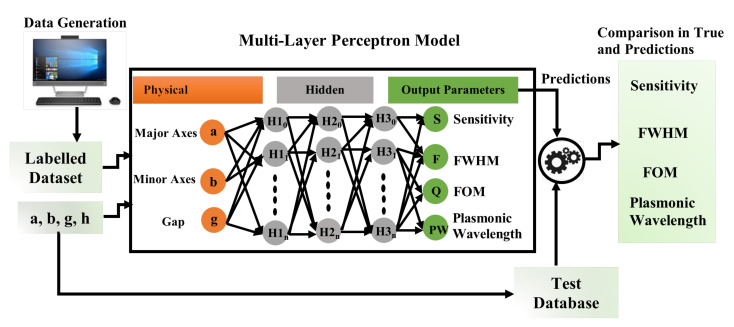
Outlayer of Artificial Neural Network.

ANNs have been introduced as a formidable way of analyzing the mapping between the topology and composition of arbitrary nanophotonics structures and their corresponding functional properties. It focuses on developing computer algorithms that helps to extract patterns and optimize complex data with a huge number of parameters. The novelty of forward ANNs is that multiple layers and neurons can be used to optimize performance. This artificial neural network has been developed in the computational machine having 8 GB RAM, 128 GB SSD, and macOS Big Sur (version 11.2.1 (20D74)) operating system. The virtual environment Jyupter Notebook (version 6.0.3) is used, which is a web-based interactive computing notebook environment having Python (version 3.8.3) installed in an anaconda (version 1.7.2) environment throughout the computation. This algorithm can handle the complex data input parameters developed from the primary input data without any specific thumb rule. As seen in Figure 2, this was organized in three layers, such as the input layer, an output layer, and hidden layers.

The fully interconnected input layers receive the input parameters which are to be interpreted. The requisite tasks are performed by the output layer, such as prediction and classification. A neural network is made up of a layer-by-layer arrangement of neurons (or nodes). A weighted relation connects each neuron in one layer to the neuron in the next layer. The weight $w_{ij}$ shows the frequency of the connection between the $i_{th}$ neuron in one layer and the $j_{th}$ neuron. A function weight is allocated to each neuron as an entry linearly combined (or summarised) and transferred through an activation function to achieve the output of the neurons. Finally, the user can compare the predicted output with the random test sets. The network can be represented as a black box that receives *m* inputs and producing *n* outputs [37], which is shown in Figure 2. Throughout this work, an optimised ANN model with 5 hidden layers and 50 nodes/neurons in each layer was used, as illustrated in Figure 2. These hidden layers were fully interconnected, which implies that each node/neuron in one layer is linked to the node/neuron in the next layer. To enable impartial evaluation when optimizing the ANN model parameters, 10% of data samples were randomly selected from the training dataset and allotted as the validation dataset (weights and biases). During the training procedure, the Rectified linear unit *ReLU* activation function [38] and Adam optimizer [39] were used to approximate the non-linear function and optimise the weights, respectively. After each iteration/epoch, the ANN model predicts particular outputs. The mean squared error (MSE) between the expected and actual outputs were computed, and the back-propagation phenomenon was used to update the hidden layer weights for each epoch.

The MLP computational system can contain an infinite number of hidden layers that are located between the input and output layers; however, in this work, a finite number of hidden layers has been used and the data flows in the forward direction from the input to the output layer, analogous to a feed-forward network in an MLP; however, the sensitivity, FWHM, FOM, and Plasmonic wavelength were considered as the output from the output layers. Figure 2 shows flow of the entire process of the artificial neural network, wherein the first step is to collect the labeled dataset from the COMSOL multiphysics when the inputs of the artificial neural network are considered as the major axis *a*, the minor axis *b*, and the separation gaps *g* have been assigned as the physical parameters that are used for input layers. There are customised hidden layers and neurons (or nodes) present between the input and output layers, which predict the output parameters, closer to the actual (or simulated) output. Neurons in the hidden layers are a crucial part of deciding the overall performance of the neural network. Even though these layers have no overt interaction with the outside world, they significantly influence the final output.

**Figure 3:**
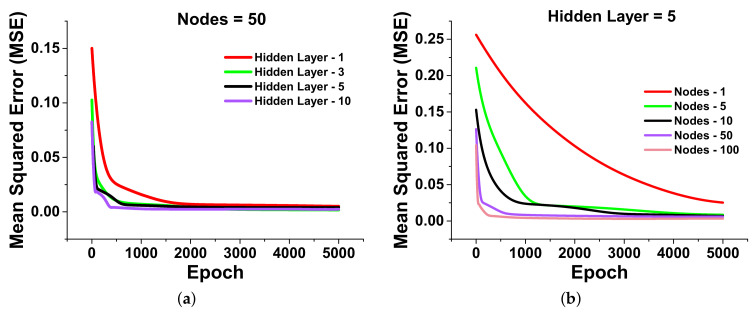
Mean squared error calculation for the above shown dataset in Table 1: (**a**) The mean squared error at different hidden layers with 50 nodes (neurons); (**b**) Neuron variation at 5 hidden layers.

The number of hidden layers and the number of neurons in each hidden layer are thoroughly researched based on mean squared errors (MSE) that can be calculated by using Equation (2):

(2) $$\begin{array}{c} MSE=\frac{1}{n}\sum_{i=1}^{n}\left(Y_{i}-Y_{i}^{p}\right) \end{array}$$

where *n* is the number of the data points used throughout process. $Y_{i}$ is defined as the actual value calculated from COMSOL multiphysics, and $Y_{i}^{p}$ represents the predicted values (e.g., from a linear regressing fit). The MSE can be estimated for each data point and the expected regression model. It is observed that the model can make accurate predictions with the lowest MSE. The actual and predicted data points of the validation and training dataset are discussed in a later section. The function of a neural network can be broken down into relevant data transformations using these hidden layers. Each hidden layer is tailored to generate a specified output. This algorithm starts from the random weights which is further tuned by varying the hidden layers (from 1 to 10) with 50 neurons and calculated the corresponding MSE as shown in Figure 3a. Here, the red curve shows the MSE = 0.14 at the first epoch for 1 hidden layer, and it decreases sharply until 1500 epochs and is stabilized for a higher epoch. However, the green curve shows MSE = 0.10 at the first epoch for 3 hidden layers and stabilized after 700 epochs.

Finally, at the first epoch, the MSEs are approximately 0.07 and 0.06 for 5 and 10 hidden layers, respectively, and after 2000 epochs, it stabilizes, as seen by the black and purple curves in Figure 3a. When the hidden layers are more than five, the MSE does not improve much; hence, five hidden layers were used for further observations. Next, the number of neurons have been varied from 1 to 100 for 5 hidden layers, as shown in Figure 3b. The MSE has been calculated as 0.25 when 1 node is used, shown by a red curve. However, MSE = 0.21 was calculated at first epoch for 5 nodes and stabilizes after 1200 epochs, shown by a green curve. The black curve shows the MSE = 0.15 for 10 nodes at the first epoch, and it stabilises after 800 epochs. In order to further reduce MSE, the algorithm for 50 and 100 epochs have also been considered, shown by purple and pink curves, respectively. In these cases, only a small change in MSE has been observed, and it became stable after 600 epochs.

Indeed, the lower the MSE, the closer the predicted regression values are to the actual values, so the model appears to be well trained with 5 hidden layers and 50 neurons, as shown in Figure 3b. This algorithm has been simulated until 5000 epochs to make sure that the MSE values decrease to their lower value. Following this investigation, 5 hidden layers with 50 neurons have been considered for all subsequent computations to reduce the computing load. After this optimization, the rectified linear activation function or ReLU has been used for designing the neural network as it is convenient, easy to use, and efficient at getting around the limitations of other popular activation functions such as Sigmoid and Tanh. It is less susceptible to vanishing gradient problems, which can prevent deep learning models from being trained. To optimise the weight’s values during the ML training process, the *Adam optimizer* was selected over LBFGS and *Stochastic Gradient Descent (SGD) optimizer* since it works well for a reasonably big dataset. When *MSE* converges to an appropriate threshold, the number of epochs to be taken is selected by the user. The appropriate outputs were provided as a new input data that was not supplied during the training procedure after tweaking the model to have a stable *MSE* value. An artificial neural network with hidden layers that uses the rectifier function is often referred to as rectified networks. ReLU implementation is undoubtedly one of the key achievements in the deep learning revolution, as the methods that now facilitate the systematic development of neural networks.

The time taken to train the artificial neural network depends on a range of factors, including the quantity of data, the number of hidden layers, the number of neurons in each hidden layer, and the number of epochs. It took about 10 s to train the designed model with the collected dataset from COMSOL, when we have used 5 hidden layers with 50 neurons in each layer and ran for 5000 epochs. The model weights and parameters were saved in the machine after the training was completed. The next step was to predict the output for unseen inputs by using previously saved weights, which took only 71 s for 5 hidden layers. On the other hand, direct numerical simulation using COMSOL multiphysics takes around 165 min, 235 min, and 636 min and a day or two for the normal, fine, finer and extremely fine meshes to calculate the sensitivity, FWHM, FOM, and Plasmonic wavelength for only one design and can take much longer if a manual mesh (smaller to extremely fine mesh size) is used.

## 3. Neural Network Analysis with Empirical Evidences

In this section, the trained artificial neural network is evaluated by assessing their predicted outputs with the actual outputs for a paired nanoparticles with random design specifications. Afterward, the predicted sensitivity, FWHM, FOM, and Plasmonic wavelength are compared with their simulated values for any the major axis *a*, minor axis *b*, and separation gap *g* values.

### 3.1. Sensitivity (nm/RIU)

**Figure 4:**
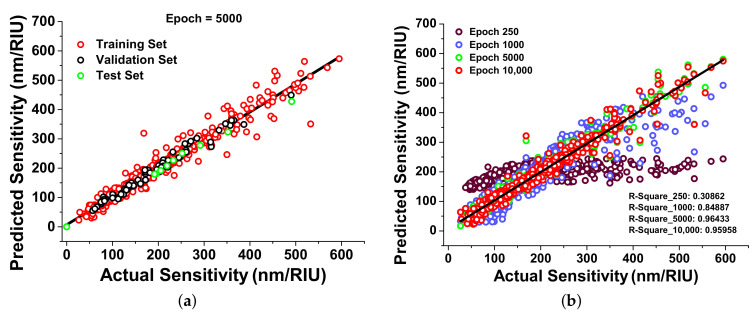
The scatter plot of a data point location: (**a**) The comparison of the training, validation, and test data set; (**b**) the efficiency of the developed neural network with epoch variance, with the comparison of actual sensitivity (nm/RIU) values (from the simulation) with respect to predicted values (calculated from the neural network).

In this section, the sensitivity [40] predictions have been observed with MSE calculation for training and validation sets at different epoch. The MSE was calculated as nearly 0.130 and 0.128 for the training and validation sets, respectively. Several new and random datasets have been adopted to observe that the model makes good assumptions on the actual datasets. Figure 4a shows the data point location of the training, validation, and test set. In Figure 4a, the red circle indicates the training dataset, which was used to train the neural network and predicts the sensitivity values for random inputs values. However, black circles display the validation dataset, which explains the accuracy of the predictions over the actual values, and green circles represent the test dataset, which is entirely unknown from the train and validation set and used to observe the accuracy of the predictions after training. This test dataset is used to validate the neural network. Each circle represents a single data point, and for a well-trained model, these values should be aligned closer to the solid black line.

Figure 4b demonstrates the improved ML model with iterations (Epoch); this graph shows the improvement in the designed neural network from 250 epochs to 10,000 epochs. To avoid over fitting, the R-Squared values have also been calculated. The predicted values of sensitivity, predicted at epoch 250 (shown by red circles), are not close to the real values shown by the solid black curve. R-Squared values have been calculated for a statistical fit that shows the variance in the predicted values as 0.31 for 250 epochs, which means the model is not yet trained properly. To improve this developed model, 1000 epochs have been adopted, and the predictions were slightly near the ideal curve, shown by the blue circles. The 0.85 R-Squared value has been calculated for 1000 epochs, which shows the improvement in predictions for neural network training. We continued increasing the epochs until becoming closer to real values, and for epochs 5000 and 10,000, the R-Squared values were calculated as 0.964 and 0.959 shown by green and red circles, respectively. As after 5000, the R-Squared value rather slightly decreased, which indicated that the model is getting over fitted as shown in Figure 4b. Hence, 5000 epochs have been selected for the further evaluations.

**Figure 5:**
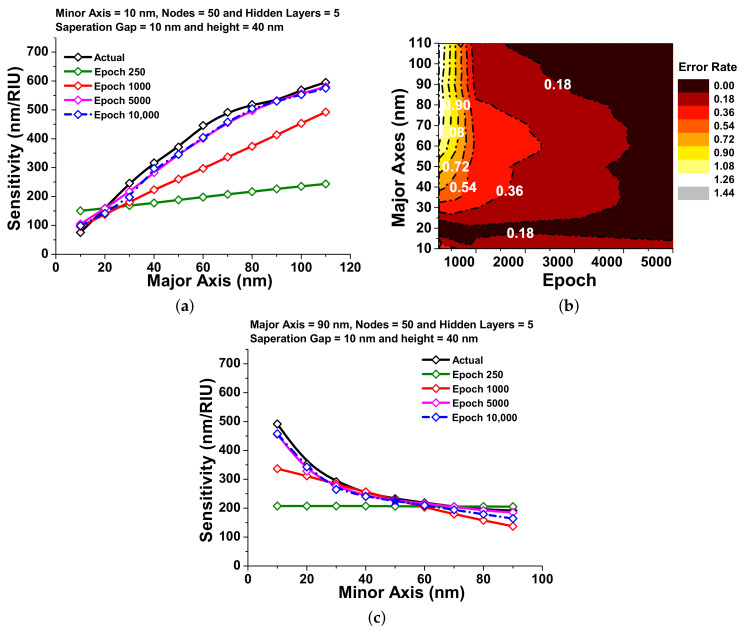
Sensitivity visualization:(**a**) the predicted sensitivity over the major axes (nm) at different epochs; (**b**) a contour plot of the absolute error values for sensitivity predictions over the major axes (nm); (**c**) the sensitivity response against the minor axes (nm) with epoch variation.

Figure 5a shows the variation of sensitivity with the major axes for different epoch values. Here, the black curve shows the actual dataset obtained from COMSOL modelling, and this dataset has been considered as a reference dataset. By changing the epoch we train the neural network in such a way that the neural network can make close predictions with respect to the reference data points. From Figure 5a, it can be observed that at 250 epochs, the difference between the actual sensitivity (shown by black curve) and predicted sensitivity (shown by green curve) is very high. However, when the neural network is trained at 1000 epochs, the difference between the predicted sensitivity (shown by a red curve) and the actual sensitivity is reduced. In order to obtain more accurate predictions over actual sensitivity, the neural network was solved for 5000 and 10,000 epochs, shown by a pink and dashed blue curves, respectively. From these comparisons, it can be seen that with a larger number of epochs, the predicted sensitivity is closer to the actual sensitivity without any over-fitting in the model. Figure 5b shows the contour plot of the error rate of the predicted sensitivity at variable major axis to optimize the absolute error values with different epoch values. It can be seen from this figure that the error rate is gradually reducing to 1.90, 1.08, 0.72, 0.54, 0.36, and 0.18 for 500, 700, 1100, 1500, 2500, 3000, and 4500 epochs, respectively. After 4500 epochs, it is observed that the error reduces close to 0.00, which can be regarded as promising findings as shown in Figure 5b, hence the trained model weights can now be saved for future evaluation at 5000 epoch. Figure 5c shows the sensitivity prediction over the minor axis from 10 nm to 120 nm. In this figure, the reference data points have been plotted by a black curve. From this figure, it can be observed that as value of the epoch is increasing, the error rate between the actual and predicted sensitivity is gradually decreasing as shown by the green, red, pink, and dashed blue curves with respect to the black curve. This shows the absolute error rate of sensitivity prediction over the minor axis from 250 to 5000 epochs. It can be observed that the error rate decreases gradually from smaller epochs to larger epochs and calculated as 0.68 for 250 epochs; however, it reduces to 0.51, 0.34, and 0.17 for 1000, 2000, and 4000 epochs, respectively, and finally reaches its lowest value for 5000. Since at 5000 epochs the absolute error rate reaches the lowest value, 5000 epochs have been optimized for further observations.

### 3.2. Plasmonic Wavelength

Plasmonic resonance wavelength plays a vital role in understanding the performance of a nano-structured antenna. Hence, in this section, the response of the predicted plasmonic wavelength (nm) (from the previously developed neural network) over the actual plasmonic wavelength (calculated from COMSOL multiphysics) have been tested. The MSE values of the neural network for training and validation set were calculated as 0.15 and 0.10, respectively, and decreases sharply from the initial epoch to the 1000 epochs, which reaches its lowest value for higher epoch values.

**Figure 6:**
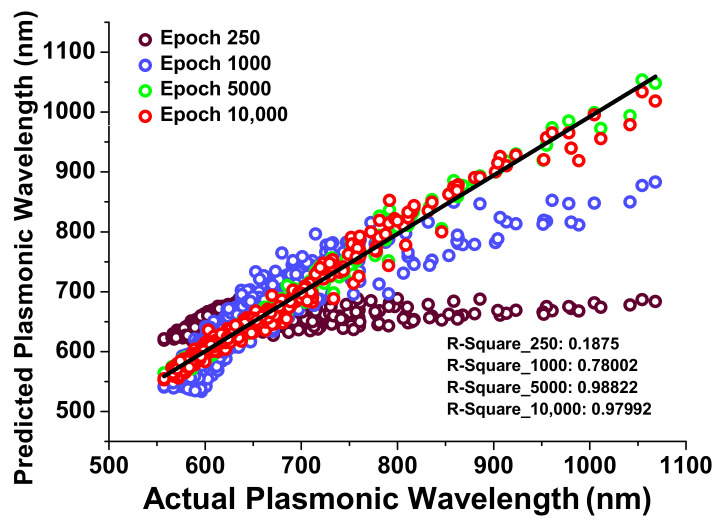
The scatter plot of a data point location shows improvement on a developed neural network with different epochs.

Figure 6 shows the improvement in the predicted plasmonic wavelength with respect to the actual plasmonic wavelength at epochs from 250 to 10,000. Here, a black curve shows the linear regression fit and purple hollow circles indicate the plasmonic wavelength at 250 epochs, which is not even close to the linear regression fit, which means the neural network is not properly trained. Due to this reason, the designed neural network has been trained for 1000, 5000, and 10,000 epochs, which are shown by blue, green, and red hollow circles, respectively. From this, it is clear that as the epoch values are increasing, predicted values are moving towards the linear regressing fit. To avoid the over-fitting in the neural network, the R-Squared value has also been calculated as 0.188, 0.78, 0.99, and 0.98 for 250, 1000, 5000, and 10,000 epoch values, respectively. It can be seen that for 10,000 epochs R-Squared values rather decreases, which means predicted values are moving towards over-fitting, hence 5000 epochs are considered for further calculations.

**Figure 7:**
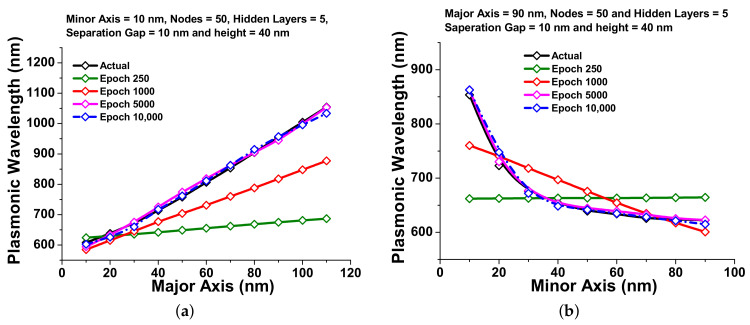
Plasmonic wavelength (nm) visualization: (**a**) the predicted plasmonic wavelength (nm) with major axes (nm) variation at different epoch; (**b**) the plasmonic wavelength (nm) response against the minor axes (nm) with epoch variation.

Next, Figure 7a shows the improvement in the predictions of the plasmonic wavelength with respect to the major axis *a*, which are compared with the actual (simulated by the finite element method) plasmonic wavelength shown by a black curve. It can be seen from this figure that the predicted plasmonic wavelength for 250 epochs, shown by a green curve is far away from the validation curve (shown by the black line), which means the neural network was not yet properly trained. Then, for 1000 epochs, the predicted values improved slightly (shown by red curve) towards the validation curve. In order to obtain the accurate predictions, the neural network has been trained for higher epochs of 5000 and 10,000, shown by pink and dashed blue curves, respectively, which has shown good accuracy with respect to the validation curve. It is clearly observed that the accuracy in the prediction increases gradually with the increment in epochs. However, this shows the absolute error values with the major axis variations which are decreasing from 250 to 5000 epoch as 0.40, 0.34, 0.27, 0.20, 0.14, and 0.069 for 300, 570, 690, 1000, 2000, and 3000 epochs, respectively. After that, it is reduced to its lowest value of 5000 epochs; due to this reason, 5000 epochs have been adopted for further calculations.

Figure 7b shows the accuracy of the prediction of the plasmonics wavelength with respect to the minor axis *b*. Here, it can be clearly observed that for lower epoch values the difference between the actual (shown by a black curve) and predicted values were high, and as the epoch increases, the accuracy in the predicted values were also increased as shown by green, red, pink, and dashed blue curves at 250, 1000, 5000 and 10,000 epochs, respectively. The absolute error values with different minor axis and epochs from 250 to 5000 were also calculated to observe the performance of the neural network. These values are 0.11, 0.073, 0.045, and 0.045 for 900, 3000, 4000, and 4300 epochs, respectively. Then, it fell to its lowest value for the 5000th epoch, and this was used for all the subsequent predictions.

**Figure 8:**
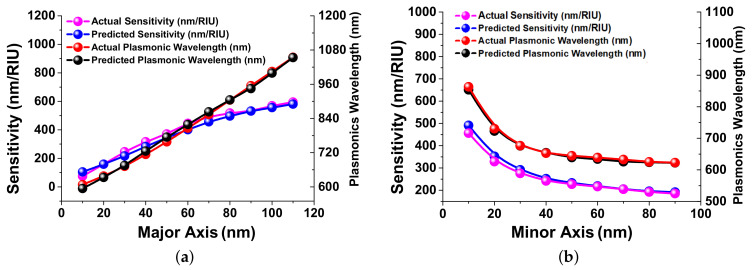
Sensitivity (nm/RIU) and Plasmonic wavelength (nm) visualization: (**a**) show actual sensitivity (nm/RIU) and plasmonic wavelength (nm) response with predicted sensitivity (nm/RIU) and plasmonic wavelength (nm) over the major axis (nm); (**b**) the difference between actual predicted sensitivity (nm/RIU) and plasmonic wavelength (nm) and predicted sensitivity and (nm/RIU) plasmonic wavelength (nm) over the minor axis (nm).

The final observations of predicted sensitivity (Left *y*-axis) and plasmonic wavelength (Right *y*-axis) with respect to major axis (*x*-axis) variation at 5000 epochs have been shown in Figure 8a. This plot presents the variation of sensitivity with the major axis shown by pink and blue curves for actual values (obtained from COMSOL multiphysics) and predicted values (obtained from the developed neural network) by using Left *y*-axis. On the other hand, the variation of plasmonic wavelength with the major axis for the actual and predicted values are shown by red and black curves, respectively, at 5000 epochs by using the Right *y*-axis. Figure 8b shows the predicted sensitivity (Left *y*-axis) and the plasmonics wavelength (Right *y*-axis) with respect to the minor axis at 5000 epochs. From this figure, it can be observed that the difference between the actual and predicted values were very small, hence it can be stated that the developed artificial neural network is showing a good performance within 65 s, whereas COMSOL takes around 5 to 6 h. This can also be inferred that this network indicates the value of sensitivity and plasmonics wavelengths very well and consistently with less computing time/load than the direct computational approaches.

### 3.3. Full-Width Half Maximum (FWHM)

To observe the sharpness of the transmission spectra of the paired nanostructured sensors, FWHM plays an important role, hence the observations of the FWHM predictions have also been shown from the above developed neural network in this section. The MSE values for the train and validation sets for the calculation of FWHM (nm) were obtained as 0.19 and 0.11, respectively, with a sharp decay after 1000 epochs. Next, the improvement of the FWHM prediction has been calculated from 250 epochs to 10,000 epochs to improve the efficiency of the developed neural network. The additional calculations were performed for 250, 1000, 5000, and 10,000 epochs, and R-Squared values have been calculated as 0.72, 0.77, 0.846, and 0.845, respectively. As the R-Squared values for 10,000 epochs have been reduced, 5000 epochs have rather been used to optimize the entire FWHM predictions.

**Figure 9:**
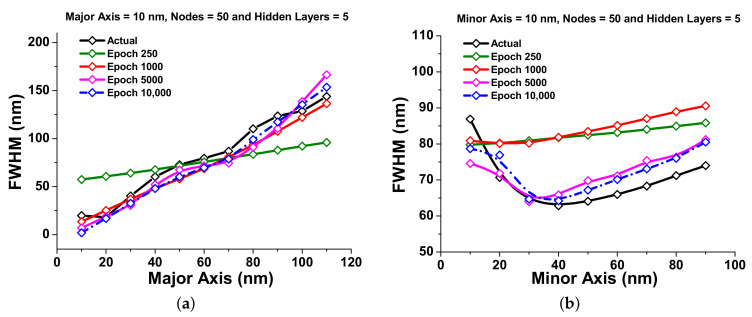
FWHM (nm) visualization: (**a**) the predicted FWHM (nm) with major axes (nm) variation at different epochs; (**b**) shows the FWHM (nm) response against the minor axes (nm) with epoch variation.

Additionally, Figure 9a shows the performance improvement of FWHM with respect to the major axis. Here, the black curve shows the reference data point obtained from COMSOL multiphysics. The FWHM predictions for 250, 1000, 5000, and 10,000 epochs have been shown by the green, red, pink, and dashed blue curves; however, the actual FWHM is depicted by a black curve. It can be seen from these curves that the number of epochs are directly related to the accuracy of the prediction until it reaches the over-fitting point, which is 5000 epochs in this case.

The error rate for the FWHM on the major axis with respect to epoch number shows a steady decrease as the epoch number is increased. For the 530, 1000, 2100, 4000, 4900, and 5900 epochs, the absolute error values were calculated as 0.61, 0.53, 0.35, 0.27, 0.18, and 0.091, respectively. The aim to calculate these values is to provide a quantitative value of the improvement in error rates. Figure 9b shows the predicted FWHM at different epochs with respect to the minor axis and compared with the actual FWHM (shown by a black curve). From this figure, it can be seen that as the epoch increases, the predicted values converges with the actual FWHM shown by green, red, pink, and dashed blue curves for 250, 1000, 5000, and 10,000, respectively. The error rate for the FWHM for different minor axes with respect to epoch is also observed to be steadily decreasing as the epoch number is increased. The error rate also reduces as the epoch number is increased, and these values are 0.2, 0.1, 0.06, and 0.03 for 3000, 3800 5000, and 8000 epochs, respectively.

## 4. Comparison of Computational and Numerical Simulations Performance

The optical transmission/reflection spectra and field distributions for the paired nanostructured antennae were obtained by using COMSOL Multiphysics software based on the finite element method (FEM). The neural network has been developed in Python for making predictions, which was discussed in the earlier section. With the help of the random input parameters, this developed neural network model made predictions on the corresponding outputs in a few seconds. However, the COMSOL multiphysics can take a longer time for generating a single parameter. In the end, we have also shown the comparison between predicted values over the actual values for random major axis, minor axis, and separation gap and its corresponding sensitivity, FWHM, and Plasmonic wavelength has also been calculated within the range (given dataset) as shown in Table 2. This also shows the absolute error rate in % between the predicted and simulated sensitivity for the random dataset. The calculated absolute error values were calculated to lie between 3.39 and 0.02%, which can be considered as good response of the in-house developed algorithm for making such close predictions.

The ANN has the distinct benefit of calculating optical parameters in a very short amount of time and with very few resources. Another advantage of the developed ANN is that it requires less formal statistical training and can discover complicated nonlinear correlations between dependent and independent variables without explicit training. In comparison to traditional regression analysis [41], this study displays the capacity to detect all potential interactions between predictor variables and offers multiple input training. Traditional regression analysis necessitates extensive statistical training and an understanding of a range of statistical concepts, such as backward and forward stepwise regression, *p* values, odds ratios, multicollinearity, interactions, and input output terms.

## 5. Conclusions

In conclusion, machine learning algorithms have been developed and used to predict the key properties of a paired gold nanoantenna for multiple input/output parameters. This paper demonstrates the crucial steps for rigorous testing of the artificial neural network and made good predictions with a trained network. Throughout the neural network, 5 hidden layers of 50 neurons each were used to provide fast convergence and high accuracy in predicting outputs for random input geometric dimensions of the nanoantennae. The MSE has also shown against the number of epochs while predicting the sensitivity, FWHM, FOM, and plasmonic wavelength for any random input parameters of different major axis, minor axis, and separation gap. This paper also shows the comparison in computational time of COMSOL multiphysics and in-house developed neural networks, which is almost achieved fives times smaller than the direct simulations. Finally, the performance of the developed model has been demonstrated for the random input parameter and predicted the corresponding output parameters. Hence, we believe that the convergence between artificial intelligence and nanotechnology could pave the way for a slew of emerging technological advances in the field of knowledge sciences. 

## Figures and Tables

**Table 1:** Dataset variation used for neural network training.

	Major Axis (nm)	Minor Axis (nm)	Gap(nm)	Sensitivity (nm/RIU)	FWHM (nm)	Plasmonic Wavelength (nm)
***count***	530	530	530	530	530	530
***mean***	89.69	49.54	39.5471	191.22	78.54	653.64
***Standard Deviation***	24.74	29.22	22.35	109.43	37.16	86.97
***Minima***	30.00	10.00	10.00	26.52	2.90	557.35
***Maxima***	130.00	130.00	80.00	595.04	202.40	1068.24

**Table 2:** Comparison between actual and predicted values within the range.

Input Parameters			Simulated Data from COMSOL Multiphysics				Predicted Data from Artificial Neural Network				Abs Error
***Major*** ***Axes*** ***(nm)***	***Minor*** ***Axes*** ***(nm)***	***Gap*** ***(nm)***	***Sensitivity*** ***(nm/RIU)***	***FWHM*** ***(nm)***	***FOM***	***Plasmonic*** ***Wavelength*** ***(nm)***	***Sensitivity*** ***(nm/RIU)***	***FWHM*** ***(nm)***	***FOM***	***Plasmonic*** ***Wavelength*** ***(nm)***	***Sensitivity*** ***(nm/RIU)*** ***%***
60	20	40	147.2138	47.5796	12.9362	615.5012	146.9226	48.2208	12.7551	614.5826	0.19
80	30	80	163.8554	70.5513	8.8533	624.6127	163.6056	73.3072	8.5141	624.1441	0.15
85	45	25	171.4485	40.5550	17.8535	724.0533	170.1578	45.2992	16.0795	728.3898	0.75
90	50	90	135.1549	72.3698	8.4007	607.9604	135.6321	76.1116	7.9741	606.9173	0.35
100	40	60	197.3106	67.2607	9.5097	639.6299	196.4355	66.1773	9.6454	638.3086	0.44
100	40	50	208.2831	69.2660	9.2666	641.8674	208.2168	70.5940	9.0697	640.2649	0.03
100	50	90	170.0086	79.0452	7.8709	622.1600	171.1509	82.6979	7.5360	623.2143	0.67
110	50	110	193.4595	90.4005	7.1131	643.0292	194.6961	92.8461	6.9959	649.5304	0.64
115	25	25	307.8743	100.8810	7.7953	786.4027	307.7973	100.0813	7.8181	782.4466	0.02
120	60	90	202.0654	125.9891	5.1380	647.3321	202.4991	118.7540	5.4480	646.9770	0.21
120	50	110	231.9397	105.6556	6.3215	667.9105	224.0618	105.1861	6.3483	667.7588	3.39
120	60	120	208.3476	105.9363	6.1832	655.0344	208.6262	104.3657	6.2874	656.1949	0.13

## Data Availability

The data is available on reasonable request from the corresponding author.
